# Supplementary material for: The causal relationship between the human gut microbiota and pyogenic arthritis: a Mendelian randomization study
Source: Front Cell Infect Microbiol. 2024 Nov 26;14:1452480. doi: 10.3389/fcimb.2024.1452480 (PMC11629706; doi:10.3389/fcimb.2024.1452480)
Supplement: Supplementary file 1 [file DataSheet1.zip › mendelian test/ebi-a-GCST90027616.csv_leave_one_out.pdf]

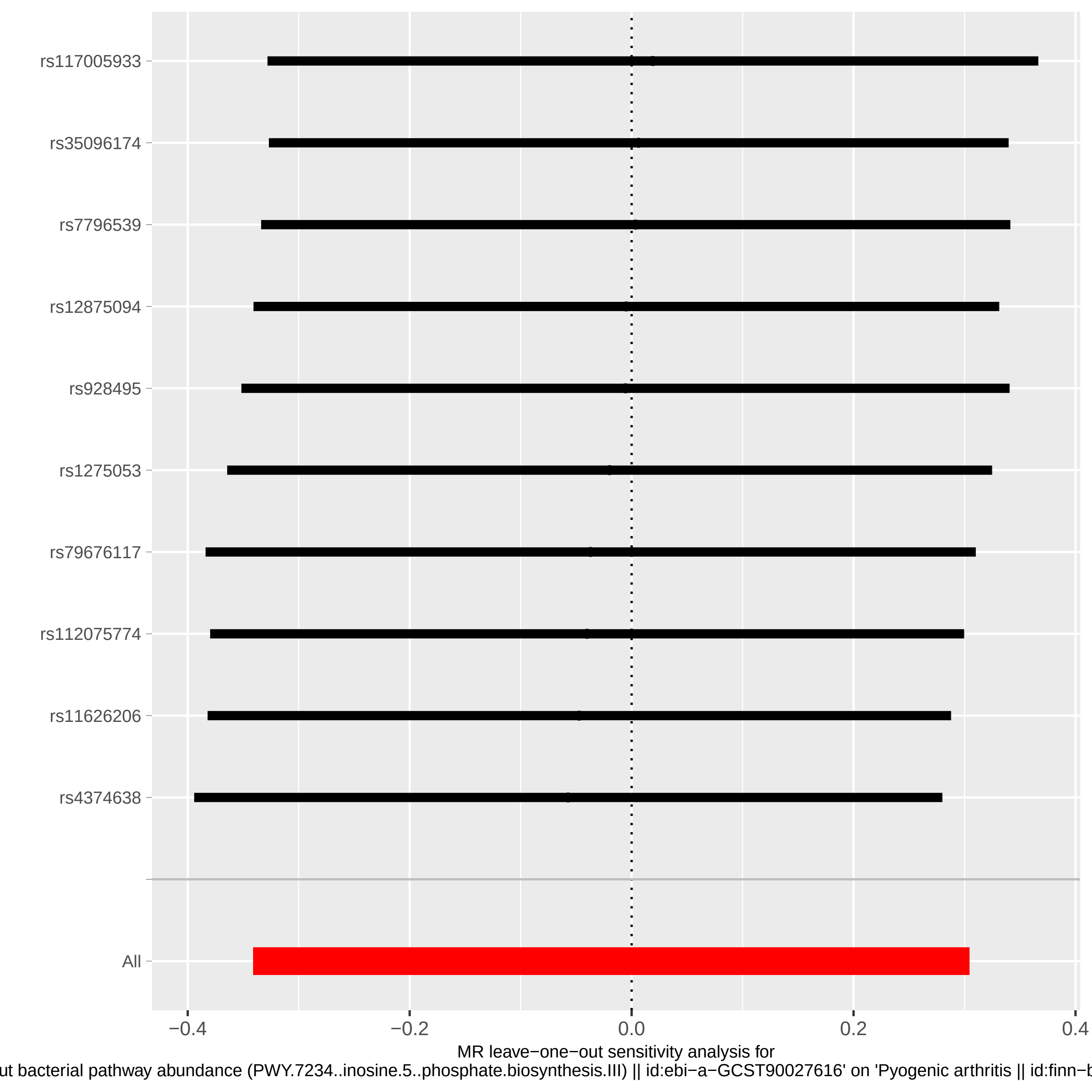
rs117005933
rs35096174
rs7796539
rs12875094
rs928495
rs1275053
rs79676117
rs112075774
rs11626206
rs4374638
All
−0.4
−0.2
0.0
0.2
0.4
MR leave−one−out sensitivity analysis for
ut bacterial pathway abundance (PWY.7234..inosine.5..phosphate.biosynthesis.III) || id:ebi−a−GCST90027616' on 'Pyogenic arthritis || id:finn−b
